# Hypertension and alcohol: a cross-sectional study comparing PEth with AUDIT and AUDIT-C in primary care

**DOI:** 10.1093/fampra/cmaf097

**Published:** 2026-01-09

**Authors:** Åsa Thurfjell, Maria Hagströmer, Charlotte Ivarsson, Anders Norrman, Johanna Adami, Lena Lundh, Jan Hasselström

**Affiliations:** Department of Neurobiology, Care Sciences and Society, Division of Family Medicine and Primary Care, Karolinska Institutet, 141 83 Huddinge, Sweden; Academic Primary Health Care Centre, Region Stockholm, 104 31 Stockholm, Sweden; Academic Primary Health Care Centre, Region Stockholm, 104 31 Stockholm, Sweden; Department of Neurobiology, Care Sciences and Society, Division of Physiotherapy, Karolinska Institutet, 141 83 Huddinge, Sweden; Sophiahemmet University, 114 86 Stockholm, Sweden; Academic Primary Health Care Centre, Region Stockholm, 104 31 Stockholm, Sweden; Department of Neurobiology, Care Sciences and Society, Division of Family Medicine and Primary Care, Karolinska Institutet, 141 83 Huddinge, Sweden; Academic Primary Health Care Centre, Region Stockholm, 104 31 Stockholm, Sweden; Sophiahemmet University, 114 86 Stockholm, Sweden; Department of Neurobiology, Care Sciences and Society, Division of Family Medicine and Primary Care, Karolinska Institutet, 141 83 Huddinge, Sweden; Academic Primary Health Care Centre, Region Stockholm, 104 31 Stockholm, Sweden; Department of Neurobiology, Care Sciences and Society, Division of Family Medicine and Primary Care, Karolinska Institutet, 141 83 Huddinge, Sweden; Academic Primary Health Care Centre, Region Stockholm, 104 31 Stockholm, Sweden

**Keywords:** alcohol use disorder, hypertension (high blood pressure), primary care, screening, prevention, cardiovascular disorders (hypertension/DVT/atherosclerosis)

## Abstract

**Background:**

This cross-sectional study aimed to describe proportions of patients with indications of alcohol consumption using phosphatidylethanol (PEth), the Alcohol Use Disorders Identification Test (AUDIT), and its consumption-focused version (AUDIT-C), in relation to blood pressure (BP) control, overall and by sex.

**Methods:**

A total of 270 hypertensive primary care patients (ICD-10: I10.9) were stratified into BP control groups: controlled (<140/90 mmHg), uncontrolled (≥140/90 mmHg), and apparent treatment-resistant hypertension (aTRH; ≥140/90 mmHg with ≥3 antihypertensive drugs). A randomized sample from each stratum was invited, baseline data were collected. Alcohol consumption using predefined categories for PEth and AUDIT, and hazardous use (PEth ≥ 0.122 µmol/L; AUDIT ≥ 8; AUDIT-C ≥ 5 for men, ≥4 for women), were analyzed in relation to BP control groups.

**Results:**

Mean age was 67 ± 11 years; 42% were women. PEth indicated high and regular alcohol consumption in 6.4% of controlled, 5.3% of uncontrolled, and 19.2% of aTRH patients (controlled vs. aTRH, *P* = .027; uncontrolled vs. aTRH, *P* = .013). AUDIT showed no significant differences in hazardous use between BP groups (*P* = .865). AUDIT-C identified slightly higher proportions of hazardous use than PEth, across BP groups and sexes. No significant differences were found between BP groups for hazardous use by PEth (*P* = .339) or AUDIT-C (*P* = .150).

**Conclusions:**

PEth revealed significantly higher alcohol use in the aTRH group, undetected by AUDIT. AUDIT-C and PEth identified more hazardous use than AUDIT, suggesting their potential to prompt alcohol-related discussions and support evidence-based hypertension care. PEth correlated more strongly with AUDIT-C than with AUDIT.

**Clinical trial registration:**

Retrospectively registered in Clinical Trials, SLSO2022-0143, 2022-12-10.

Key message

- PEth indicated higher alcohol use in patients with aTRH patients than AUDIT.
- AUDIT-C and PEth identified more hazardous use than AUDIT.
- No BP group differences in hazardous use by PEth or AUDIT-C.
- PEth correlated more strongly with AUDIT-C than with AUDIT.
- Alcohol screening may improve hypertension management in primary care.

## Background

Hypertension and harmful alcohol consumption are the second and ninth most common risk factors for the global burden of diseases, respectively [1]. Hazardous alcohol use [2] is associated with increased risk of some noncommunicable diseases [3]. Alcohol consumption generally increases blood pressure (BP) [4] and is not exclusively related to chronic high consumption or binge drinking [5]. It is recommended to increase screening for hazardous alcohol use in newly diagnosed and uncontrolled hypertension within primary care [6].

Treatment guidelines for elevated BP and hypertension emphasize lifestyle interventions as a part of first-line therapy, including the moderation of alcohol consumption [7]. Hypertension is primarily treated within primary care settings [8]. However, general practitioners (GPs) seldom address alcohol in the management of hypertension [9] and advice about alcohol seems to be given less often than for other unhealthy lifestyles factors [10]. In addition, hazardous and harmful alcohol use may be an important factor in partly explaining uncontrolled and apparent treatment-resistant hypertension (aTRH), commonly presenting as clinically problematic situations although seldom addressed [7, 11].

A particular focus in primary care today is to rule out whether the patient has hazardous alcohol use or not [6]. This at-risk group may include patients who drink a certain amount of alcohol, those with harmful alcohol use, or with alcohol dependence [2]. There is, however, no internationally harmonized definition of hazardous alcohol use [2]. Several guidelines though, define hazardous alcohol use as consuming more than 98 to 280 g of pure alcohol per week [2]. In Sweden the definition is ≥120 g of pure alcohol/week or ≥4 standard units (SU) of 12 g pure alcohol at the same occasion [12].

The questionnaire Alcohol Use Disorders Identification Test (AUDIT) is recommended in primary care to identify tentatively hazardous alcohol use [13] but there are indications that AUDIT is underutilized in primary care due to factors like lack of time [14]. The shorter Alcohol Use Disorders Identification Test Consumption (AUDIT-C) is regarded more time efficient [15]. However, patients may underreport the consumption when using questionnaires [16] due to factors like social desirability [17].

An AUDIT score of ≥8, the established cut-off for hazardous alcohol use [13], correspond to an average daily consumption of over 60 g of alcohol for men (420 g per week) and over 40 g of alcohol for women (280 g per week) [18]. An AUDIT-C score of ≥4 for women and ≥5 for men indicate hazardous alcohol use [15]. For women, this equates to a weekly intake of 14 SU, each containing 8 g of pure alcohol (totaling 112 g), whereas for men, the corresponding weekly intake is 21 SU (168 g of pure alcohol) [15].

Common indirect alcohol biomarkers, such as mean corpuscular volume and gamma-glutamyl transferase, have lower sensitivity and specificity compared to direct alcohol biomarkers [19]. The direct alcohol-specific biomarker Phosphatidylethanol 16:0/18:1 (PEth) [20] is another option to identify tentatively hazardous alcohol use in health care including primary care [20–23]. GPs who used PEth in connection with hypertension perceived PEth as a test providing a reliable picture of alcohol consumption [23]. Due to significant interindividual variations, the specific PEth value corresponding to exact alcohol consumption remains unclear [21] as well as the cut-off for PEth corresponding to hazardous alcohol use [24–27]. However, guidelines provide direction for interpreting PEth values (µmol/L) in relation to three generally described categories of alcohol consumption: “no/low/sporadic” (PEth <0.05), “low to high” (PEth 0.05–0.30), and “indicates regular, high” (PEth >0.30) [21, 28]. In a population based, longitudinal cohort study (*n* = 24.574) [27], PEth (µmol/L) was related to self-reported alcohol consumption and CAGE (Cut-down, Annoyment, Guilt Eyeopener) questionnaire [29]. This cohort study proposed PEth thresholds of ≥0.057 µmol/L for average daily consumption exceeding one SU (12 g of pure alcohol), ≥0.087 µmol/L for more than two units, and ≥0.122 µmol/L for more than three units per day [27].

There is a lack of studies comparing different levels of alcohol consumption as indicated by the clinically used methods PEth 16:0/18:1, the questionnaires AUDIT, and AUDIT-C in relation to BP control. The aim of this cross-sectional study was to describe proportions of patients with indications of different alcohol consumption, including hazardous alcohol use, using these three methods in relation to BP control (in general <140/90 mm Hg) [7], for all and by sex.

## Method

### Design

This observational cross-sectional study with a stratified and randomized selection included 270 patients with hypertension at three Primary Health Care Centers (PHCCs) representing populations with varying socioeconomic characteristics in the Stockholm Region, Sweden from March 2022 to May 2024.

### Sample

The data extraction tool Medrave4 [30] was used to identify all patients registered with the PHCCs, aged 30 to 85 years, and diagnosed with essential hypertension (ICD-10 code I10.9) over the past 2 years. The age range was chosen to suit the idea of the stratification applied, as individuals under 30 more often present with secondary hypertension while those over 85 needs an individualized approach [4, 7]. Patients were excluded if they were not registered at one of the three PHCCs at the time for inclusion, had protected personal identity numbers or locked medical records, were unable to visit the PHCC, were unable to independently answer digital questions (due to dementia, severe mental illness, or the need for an interpreter), or had a recent hypertension check-up within the past nine months in adherence to clinical routine. Another exclusion criterion was secondary hypertension, ICD codes: O13.9 (gestational), I15.0 (renovascular), I15.1 (other renal disorders), I15.2 (endocrine disorders), I15.8 (other specified secondary hypertension), and I15.9 (unspecified).

**Figure 1:**
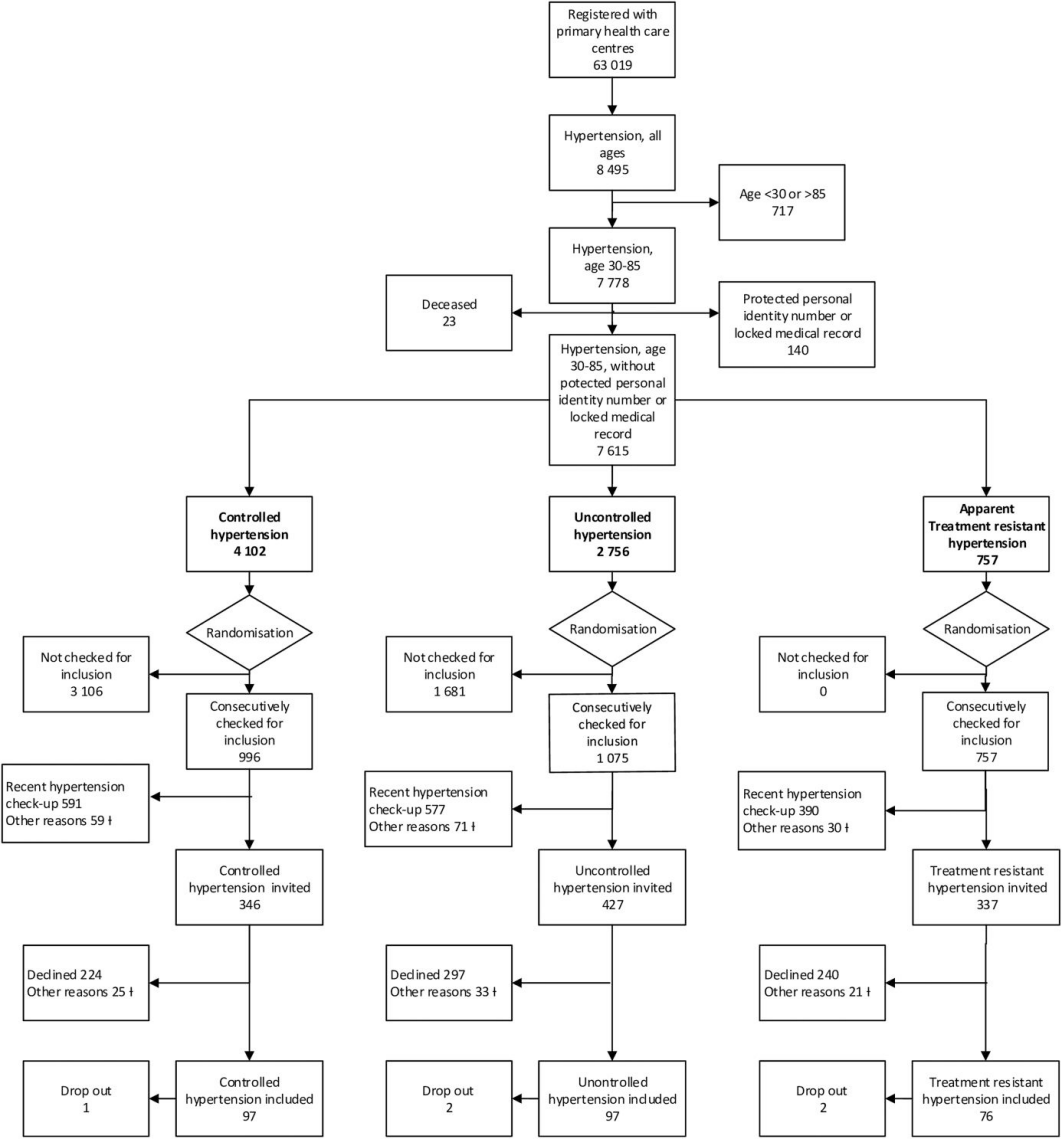
Flowchart of inclusion process and study population divided into three strata: controlled hypertension <140/90 mmHg, uncontrolled hypertension ≥140/90 mmHg, and apparent treatment-resistant hypertension ≥140/90 mmHg with at least three antihypertensive drugs of any class. † A detailed description of exclusions and “other reasons” in flow chart is found in Supplementary file S1, Supplementary Table S1.

(i) Based on the latest BP and the number of prescribed classes of antihypertensive drugs, all patients with essential hypertension were stratified into three groups of BP control: controlled (<140/90 mmHg) [7], uncontrolled (≥140/90 mmHg) [7], and aTRH (≥140/90 mmHg with at least three classes of antihypertensive drugs regardless of class [31]). (ii) Subsequently, the three BP control groups were randomized separately using the RANDOM function in Microsoft Excel. (iii) Patients in the three BP control groups were thereafter consecutively checked for inclusion, by the study nurse, to obtain a sample estimated to be sufficiently large to eventually include 90 patients from each BP control group. Due to the small group with aTRH, all individuals were screened for eligibility, resulting in 76 included patients. To meet the required sample size (*n* = 270) from the power calculation, an additional 97 participants were recruited from the controlled and uncontrolled BP groups. (iv) Patients who met the inclusion and exclusion criteria were invited to participate. Participants were informed about the full study protocol, including PEth testing, and enrolled only after giving written consent. Recruitment continued until 270 participants were recruited from all three PHCCs. A flow chart of the inclusion process and study population is presented in Fig. 1, complemented by a more detailed description in Supplementary file, Supplementary Table S1.

### Procedure

Data were collected at baseline using Research Electronic Data Capture (REDCap) [32] hosted by Karolinska Institutet. The study nurse collected data on sex, age, BP, body mass index, pulse, and adherence to antihypertensive drug classes. BP was measured with the semi-automatic professional blood pressure monitor Omron HBP-1320. Participants were seated, with BP measured in the right arm if possible, using an appropriately sized cuff. Measurements were taken twice after 5 minutes of rest, with 1-minute intervals. Exact values were recorded, and the mean value calculated. If an irregular pulse was detected, BP was measured manually with a sphygmomanometer.

During the visit, participants answered questions digitally in REDCap, see Supplementary file. The data consisted of questions on comorbidity, lifestyle habits (tobacco including smokeless tobacco “Swedish snus,” physical activity, and food) [33], the Swedish version of AUDIT [13], quality of life [34], and a selection of sociodemographic questions from The National Public Health Survey in Sweden. Additionally, data on comorbidity ICD-10 codes were extracted from the medical record with MedRave4 [30] Comorbidity data extracted from medical records was considered more reliable than self-reported information and was therefore exclusively used in the analysis. The study nurse reviewed the participants' questionnaires and, if necessary, clarified any misconceptions with the participants. In 22 AUDIT forms, the response to question 1 and questions 3 to 10 was “never,” while the response to question 2 was “missing.” In these forms, the “missing” responses were changed to “never.” In one form, the response to question 1 and questions 4 to 10 was “never,” while the response to questions 2 and 3 was “missing.” In this form, the “missing” responses were changed to “never.” In 11 forms, the response to question 1 was “never,” and the response to questions 2 to 10 was “missing.” These “missing” responses were changed to “never.”

Routine laboratory tests in connection to hypertension and PEth was analyzed using national standard assays at accredited laboratories utilized by each PHCC. Measurement of B-PEth 16:0/18:1 in EDTA whole blood was performed using liquid chromatography–tandem mass spectrometry (LC-MS/MS) at Unilabs (Eskilstuna, Sweden) and Synlab (Täby, Sweden), essentially as previously described [35]. The estimated glomerular filtration rate (eGFR) was calculated using the revised Lund-Malmö formula [36]. Participants completed the AUDIT form during their visit with the study nurse and were encouraged to take lab tests within 2 weeks. If unhealthy lifestyle habits were noted, the nurse provided advice and offered support. The visits were free of charge. Participants later visited their GP for an out-of-study annual hypertension check-up and received lab results.

### Definitions of alcohol consumption with PEth, AUDIT, and AUDIT-C

The rationale for comparing PEth, AUDIT and AUDIT-C in this study is their current use in primary care to assess indications of patients' alcohol consumption. To match guidelines [2], hazardous alcohol use with PEth (Hazardous alcohol use^PEth^) was defined as ≥0.122 µmol/L [27]. Hazardous alcohol use ^AUDIT^ was defined as ≥8 points [13] and hazardous alcohol use ^AUDIT-C^ as ≥4 points for women an ≥5 points for men [15]. In addition to hazardous alcohol use, three levels of indicated alcohol consumption were analyzed using both PEth and AUDIT. Table 1 presents all values of PEth (µmol/L) and the AUDIT and AUDIT-C scores (points) used in the study.

**Table 1:** Values of PEth, AUDIT, and AUDIT-C used in the study of alcohol consumption in 270 Swedish primary care patients with hypertension (ICD-10 code I10.9), 2022–2024.

PEth µmol/L Established interpretation guidelines [21, 28]	Interpretation [21, 28]	Approximate equivalents for standard units (SU) of 14 g pure alcohol [28]
**<0.05**	No/low/sporadic alcohol consumption	No consumption to an average of less than two SU per day
**0.05–0.3**	Low to high alcohol consumption	An average of two to four SU per day on multiple days per week.
**>0.30**	Regular and high alcohol consumption	At least four SU per day on multiple days per week.
**PEth µmol/L** **Suggested cut-off [27]**		Equivalents for standard units (SU) of 12 g pure alcohol [27]
**≥0.122**		More than three SU per day
**AUDIT points [13]**	Interpretation [13]	
**0–7**	No hazardous alcohol use	
**8–15**	Hazardous alcohol use	
**16–40**	High level of alcohol problem	
**AUDIT-C points, women [15]**	Interpretation [15]	Equivalents for standard units (SU) of 8 g pure alcohol [15]
**≥4**	Hazardous alcohol use	More than 14 SU per week
**AUDIT-C points, men [15]**		
**≥5**	Hazardous alcohol use	More than 21 SU per week

### Statistical analyses

The sample size was based on a power calculation. Based on the priori value from [37] the required sample size given an effect size 21%, significance level 5%, and power 80% using contingency tables with 2 degree of freedom will require n = 215 patients. To safeguard against potential underpowering, a total of 270 participants were included. Alcohol consumption in relation to BP control were analyzed using contingency tables (PEth, AUDIT, or AUDIT-C and BP control groups) and chi-square tests, unless otherwise stated. Analyses were performed for all participants and separately by sex. Baseline characteristics for continuous data were calculated as means with 95% confidence intervals (CI), and proportions for categorical data. Group comparisons used one-way ANOVA for means and Chi-squared tests for categorical data. Quality of life, measured with the Visual Analog Scale, was analyzed using the non-parametric Kruskal–Wallis test. A *P*-value < .05 was considered significant. Data were analyzed with IBM SPSS Statistics 26.

## Results

Baseline characteristics of participants are described in Table 2. The mean age of all participants was 67 ± 11 years, with 42% women. Mean systolic BP and diastolic BP measured in the study was 137.0 ± 16 mmHg and 83 ± 10 mmHg, respectively.

**Table 2:** Baseline characteristics of 270 Swedish primary care patients with hypertension (ICD-10 code I10.9) in relation to blood pressure control (2022–2024).

	Controlled hypertension^a^ *n* = 97 35.9%	Uncontrolled hypertension^b^ *n* = 97 35.9%	Apparent treatment resistant hypertension^c^ *n* = 76 28.1%	All *n* = 270	*P*
**Data from medical record**					
**Systolic blood pressure, mmHg (mean ± SD)**	126 ± 11	145 ± 13	148 ± 11	138 ± 15	.**000^o^**
**Diastolic blood pressure, mmHg (mean ± SD)**	78 ± 7	89 ± 9	85 ± 10	83 ± 10	.**000^o^**
**Diabetes mellitus^d^, % (*n*)**	8.2 (8)	12.4 (12)	18.4 (14)	12.6 (34)	.134^n^
**Coronary heart diease^e^, % (*n*)**	9.3 (9)	3.1 (3)	13.2 (10)	8.1 (22)	.**049**^n^
**Atrial fibrillation^f^, % (*n*)**	7.2 (7)	5.2 (5)	7.9 (6)	6.7 (18)	.745^n^
**Congestive heart failure^g^, % (*n*)**	4.1 (4)	2.1 (2)	3.9 (3)	3.3 (9)	.683^n^
**Cerebrovascular injury^h^, % (n)**	2.1 (2)	0.0 (0)	1.3 (1)	1.1 (3)	.383^n^
**Data collected in connection with study nurse**					
**Female, % (n)**	48.5 (47)	46.4 (45)	28.9 (22)	42.2 (114)	.**021^n^**
**Age, years (mean ± SD)**	65 ± 11	67 ± 10	69 ± 11	67 ± 11	.**029^o^**
**Systolic blood pressure, mmHg (mean ± SD)**	133 ± 15	138 ± 14	141 ± 17	137 ± 16	.**006^o^**
**Diastolic blood pressure, mmHg (mean ± SD)**	82 ± 9	85 ± 10	83 ± 11	83 ± 10	.121^o^
**fP-Glucose, mmol/L (mean ± SD)**	5.9 ± 1.0 (1 missing)	5.8 ± 0.0 (2 missing)	6.3 ± 1.2 (2 missing)	6.0 ± 1.0 (5 missing)	.**026^o^**
**Non-HDL cholesterol, mmol/L (mean ± SD)**	3.5 ± 1.1 (1 missing)	3.4 ± 0.9 (2 missing)	3.2 ± 1.0 (2 missing)	3.4 ± 1.0 (5 missing)	.074^o^
**eGFR < 60 ml/min/1,73 m², % (*n*)**	17.2 (16) (4 missing)	8.4 (8) (2 missing)	27.4 (20) (3 missing)	16.9 (44) (9 missing)	.**005^n^**
**Basic education^i^, % (*n*)**	17.5 (17)	9.3 (9)	17.3 (13) (1 missing)	14.5 (39) (1 missing)	.241^n^
**Medium education^j^, % (*n*)**	34.0 (33)	47.4 (46)	37.3 (28) (1 missing)	39.8 (107) (1 missing)	.241^n^
**High education^k^, % (*n*)**	48.5 (47)	43.3 (42)	45.3 (34) (1missing)	45.7 (123) (1 missing)	.241^n^
**Living with partner/husband/wife, % (*n*)**	67.0 (65)	58.8 (57)	56.6 (43)	61.1 (165)	.316^n^
**Living alone, % (*n*)**	24.7 (24)	36.1 (35)	35.5 (27)	31.9 (86)	.171^n^
**Single with children, % (*n*)**	4.1 (4)	3.1 (3)	5.3 (4)	4.1 (11)	.773^n^
**Antihypertensive drugs used (mean ± SD)**	1.6 ± 0.8	1.5 ± 0.7	2.9 ± 0.7	1.9 ± 0.9	.**000^o^**
**Overweight/obesity^l^, % (*n*)**	70.1 (68)	75.0 (72) (1 missing)	81.3 (61) (1missing)	75 (201) (2 missing)	.241^n^
**Quality of life^m^ (Med ± SD)**	80 ± 18 (2 missing)	81 ± 16 (2 missing)	75 ± 20 (1 missing)	80 ± 18 (5 missing)	.061^p^

Bold indicates *P* < .05.

^a^SBP <140 and DBP <90 mmHg.

^b^SBP ≥140 and/or DBP ≥90 mm Hg.

^c^SBP ≥140 and/or DBP ≥90 mm Hg with at least three antihypertensive medications regardless of class.

^d^ICD-code E10- E14.

^e^ICD-codeI20-I25.

^f^ICD-code I48.

^g^ICD-code I50.

^h^ICD-code I61-I64.

^i^Primary-, elementary-, or secondary-school.

^j^Upper secondary school.

^k^University.

^l^BMI ≥25 kg/m2.

^m^EQ-5D-5L VAS. HDL, High Density Lipoprotein eGFR; Estimated Glomerular Filtration Rate.

^n^Proportion of variable in relation to blood pressure control groups, Pearson Chi-Square.

^o^Mean of variable in relation to blood pressure control groups, One way ANOVA.

^p^Median of variable in relation to blood pressure control groups, Kruskal-Wallis.

**Figure 2:**
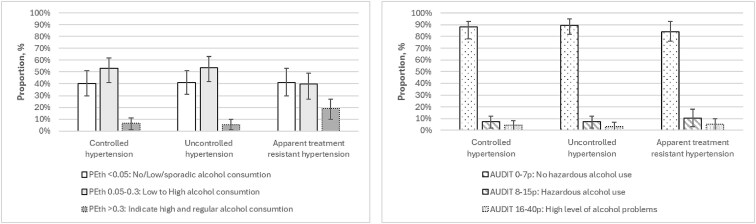
Prevalence of three categories of alcohol consumption as indicated with PEth values (µmol/L) and AUDIT (points), in relation to blood pressure control groups, AUDIT (*P* = 0.865), PEth (*P* = 0.018).

The prevalence of three categories of alcohol consumption, based on PEth values (µmol/L) [21] and AUDIT (points) [13], in relation to BP control groups is depicted in Fig. 2. Based on PEth, the prevalence of “high and regular” alcohol consumption was approximately three times higher in the group with aTRH compared to those with controlled hypertension (*P* = .027) and also significantly higher compared to those with uncontrolled hypertension (*P* = .013). Among men, a significant difference in the distribution of alcohol consumption levels based on PEth was observed between BP control groups (*P* = .018), whereas no such difference was found among women. With AUDIT, the majority in the BP control groups had not hazardous alcohol use. There were no significant differences of the distribution of different levels of alcohol consumption (no hazardous alcohol use, hazardous alcohol use, high level of alcohol problems), based on AUDIT across the three BP control groups (*P* = .865). No significant difference in the distribution of alcohol consumption levels based on AUDIT was observed between BP control groups for either men or women.

In the entire study population, 40.8% had “no/low/sporadic consumption,” 49.6% had “low to high consumption,” and 9.5% had “regular and high consumption” based on PEth. Based on AUDIT, 87.6% had no hazardous use, 8.3% had hazardous use, and 4.1% had high level of alcohol problems.

No significant difference was observed between men and women in the distribution of alcohol consumption levels based on PEth (*P* = .158). However, a significant difference was found in the distribution based on AUDIT (*P* = .013).

**Figure 3:**
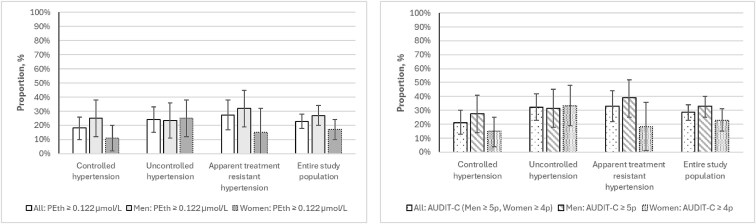
Prevalence of hazardous alcohol use^PEth^ and hazardous alcohol use^AUDIT-C^ in blood pressure control groups and in the entire study population, including men and women separately.

The prevalence of Hazardous alcohol use^AUDIT-C^ was higher compared to hazardous alcohol use^PEth^ for the entire population, in the three BP control groups, and among both men and women, see Fig. 3. There were no significant differences in the prevalence of hazardous alcohol use^PEth^ between the BP control groups (*P* = .339) or for Hazardous alcohol use^AUDIT-C^ (*P* = .150). The same was true for men (PEth *P* = .576 and AUDIT-C *P* = .469) and women (PEth *P* = .199 and AUDIT-C *P* = .092). Within each BP control group, there was no significant difference between men and women regarding hazardous alcohol use^PEth^ neither for hazardous alcohol use^AUDIT-C^. The differences between men and women for hazardous alcohol use across the entire study population were not significant for hazardous alcohol use^PEth^ (*P* = .065) or hazardous alcohol use^AUDIT-C^ (*P* = .071).

A Spearman rank correlation coefficient of 0.590 was observed between PEth and AUDIT, and 0.606 between PEth and AUDIT-C. The correlation was statistically significant (*P* < .01).

## Discussion

To our knowledge, this is the first study attempting to quantify the prevalence of different alcohol consumption in relation to three common categories of blood pressure control in primary care. This observational cross-sectional study, with a stratified (three BP control groups) and randomly selected sample of 270 primary care patients with hypertension, identified proportions of patients with indications of different levels of alcohol consumption, including hazardous alcohol use, using three established methods: the alcohol biomarker PEth, the questionnaires AUDIT, and AUDIT-C.

PEth revealed a significantly higher prevalence of high and regular alcohol use in the group with aTRH, undetected by AUDIT. Both AUDIT-C and PEth identified a higher proportion of individuals with hazardous alcohol use compared to AUDIT. While hazardous use was more frequently identified with AUDIT-C than with PEth, no significant differences were observed between BP control groups or between sexes, regardless of the method used. PEth correlated more strongly with AUDIT-C than with AUDIT.

We acknowledge that these three methods partly capture different aspects of alcohol use.

AUDIT is a validated tool to identify hazardous and harmful alcohol use and possible dependence [13] but AUDIT-C includes only questions related to consumption [15]. PEth mirrors the last 2–4 weeks of alcohol consumption despite interindividual variation [21]. Nevertheless, it is valuable to describe the proportion of patients with different levels of alcohol consumption according to each method, in order to illustrate how these tools relate to one another from a practical primary care perspective.

Alcohol raises BP in a dose-dependent pattern [4, 38]. In our study, the prevalence of PEth values indicating “high and regular consumption” was at least three times higher (19.2%) in the group with aTRH compared to groups with controlled and uncontrolled hypertension. In contrast, only 5.3% of the aTRH group had a high level of alcohol problems based on AUDIT. These results highlight the importance of identifying high alcohol consumption in patients with aTRH, suggesting that PEth is more sensitive than AUDIT for this purpose.

About half of the participants had alcohol consumption, with PEth, ranging from “low to high,” which can create ambiguity for both patients and health care professionals (HCPs) in assessing if alcohol is of medical concern for the individual patient as “low” corresponds to no alcohol or less than 2 SU per day (<28 g), while “regular and high” corresponds to at least 4 SU per day (56 g) on multiple occasions per week according to PEth interpretation guidelines [21, 28].

Compared to AUDIT, both AUDIT-C and PEth identified a higher prevalence of hazardous alcohol use, possibly because AUDIT includes items on harmful use and dependence that may be more susceptible to underreporting due to social desirability bias [17]. The discrepancy between PEth and AUDIT and AUDIT-C, may partly be explained by the fact that PEth reflects alcohol consumption over the past few weeks, whereas both AUDIT and AUDIT-C relate to intake over the past year. Additionally, interindividual variability in PEth formation may contribute to the discrepancy.

As both PEth and AUDIT-C identify a higher prevalence of indicated hazardous alcohol use compared to AUDIT, there is reason to believe that these methods may prompt more frequent conversations about alcohol. In a previous qualitative study conducted in primary care [23], general practitioners described PEth as providing a more accurate representation of alcohol consumption than AUDIT, which is line with our findings. In health care settings with lack of time, AUDIT-C as become a more feasible option than the full AUDIT [15], and in primary care PEth results are perceived as enabling quicker initiation of alcohol-related discussions [23] and being time-saving [22].

Guidelines for prevention of cardiovascular disease [4] and treatment of hypertension [7] recommend a consumption of less than 100 g of pure alcohol per week [7]. Therefore, in their guiding role, HCPs need sensitive and reliable instruments to introduce a dialogue of alcohol consumption in relation to recommended limits. Based on our findings, both PEth and AUDIT-C indicated that approximately 20% of individuals in the controlled blood pressure group, and nearly 30% in the uncontrolled and aTRH groups, had alcohol consumption levels exceeding these guidelines. However, as the optimal is to avoid alcohol altogether [7] any elevated PEth value could warrant a discussion about alcohol.

PEth has been proposed for implementation different health care setting [20] including in primary care, for example, in the management of hypertension, based on its nature as an objective and alcohol-specific biomarker [39] and repeated testing may serve as a motivational tool for behavioral change through biofeedback [39]. Limited reliability of self-reported alcohol consumption may hinder alcohol-related conversations for both HCPs and patients. In cardiology settings, HCPs tend to question self-reported alcohol consumption, which is why more objective tests such as PEth may increase motivation to address alcohol use and, when appropriate, offer brief interventions [40]. Patients treated in cardiology clinics have described difficulties in quantifying their own alcohol consumption and therefore perceive advantages in using PEth as an objective alcohol biomarker [41]. PEth may advantageously be used as a complement to self-reported alcohol consumption in order to enhance diagnostic accuracy [20].

Regulating the availability, pricing, and marketing of alcohol is the most effective way to reduce alcohol-related harm at the population level [3, 42]. At the individual level, several factors influence the motivation to reduce alcohol consumption, with concern about negative health effects being among the most common [43]. Although advice from healthcare professionals is reported to be one of the least common reasons for reducing alcohol intake [43], this does not necessarily mean that such advice is insignificant for the individual and many individuals expect HCPs to address alcohol [44]. Despite modest effects [45] and implementation challenges [46], brief interventions remain recommended [47]

Patient autonomy should be respected when using alcohol biomarkers [39, 48, 49] and AUDIT [13]. Patients express a desire for transparency and respectful dialogue when PEth is used [41], and in certain situations, they may even request repeated testing to demonstrate low consumption [49]. GPs report varying experiences regarding how and when patients should be informed about PEth, in order to respect autonomy while ensuring safe care [22, 23]. Motivational interviewing is perceived as helpful when PEth results do not align with the clinical history [50].

### Strengths and limitations

Strengths of the study includes random sampling of all patients with hypertension diagnoses at each PHCC. This recruiting process likely contributed to the even distribution of non-participants across BP control groups, adding to the reliability of our results. Other strengths include the use of three validated and established methods, PEth, AUDIT, and AUDIT-C. Additionally, data collection direct into the RedCap minimized recording errors, enhancing internal validity.

However, the study is not without limitations. The three PHCCs where participants were recruited formed as a convenience sample, potentially leading to selection bias. Another source of selection bias was excluding those who had recently undergone a hypertension check-up. We included patients this way to avoid burdening the PHCCs with inviting patients who had already attended an annual check-up. About 75% of those invited did not participate, with a potential effect on external validity. However, the proportion of non-participants was similar across the BP control groups.

The stratification into three groups of BP control was based on the latest BP documented in the medical record and prescribed antihypertensive drugs, with no knowledge of method for measuring BP or adherence to antihypertensive drugs. Thus, there may be a risk that the stratification into the BP control groups may not be correct. However, we assume that BP measurements at the PHCCs were performed in accordance with clinical guidelines. We used a suggested epidemiological definition of aTRH [31], which does not exactly match the clinical concept of TRH, where most guidelines recommend out-of-office BP measurements and adherence checks of antihypertensive medication [51]. However, most patients with true TRH are likely included in our group of aTRH. A strength is that the stratified sampling enriched the population with a higher proportion of aTRH (28%), compared to the average of 14.7% [52] and therefore enabled us to study the group with aTRH without the need to include additional participants.

In the aTRH group, the proportion of men was notably higher (71.9%) compared to the controlled and uncontrolled BP groups. Typically, the sex distribution in aTRH is reported to be around 51–53% male. However, as kidney function declines, the proportion of men tends to increase, as demonstrated by Mielke et al. (2023), where 70% of patients with aTRH were male [53]. In our study, the proportion of participants with low eGFR was nearly twice as high in the aTRH group compared to those without aTRH, which may partly explain this sex imbalance. Another possible explanation may be selection bias, as we only recruited participants who had not recently undergone hypertension check-up.

Regardless of drinking habits, patients may have chosen to participate or not, potentially leading to both overestimation and underestimation of alcohol consumption. It is possible that patients with high alcohol consumption declined participation due to feelings of shame, while those with low consumption may not have considered themselves relevant for a study focusing on alcohol. To get an understanding of the clinical validity of our study we compared our study population with a hypertensive population from a PHCC, where PEth has been used for hypertension since 2016 (unpublished data). Generally, alcohol consumption, as indicated with PEth, was lower in the comparative PHCC and the proportion of women slightly higher. This suggests that our study may have overestimated high alcohol consumption. However, our study was conducted at PHCCs in a major urban city which is associated with generally higher alcohol consumption [54] while the comparative PHCC was in a smaller city.

## Conclusion

This study highlights the importance of using tests for alcohol consumption in the management of hypertension, especially in men and in the investigation of aTRH. At a threshold of 0.122 µmol/L, PEth identified fewer patients as potentially having hazardous alcohol use compared to an AUDIT-C score of ≥5 *P* for men and ≥4 points for women. Compared to PEth and AUDIT-C, AUDIT ≥ 8 identified the fewest patients with hazardous alcohol use. PEth showed a moderate correlation with AUDIT, and a slightly stronger correlation with AUDIT-C. This suggests that PEth, either alone or in combination with AUDIT and/or AUDIT-C, can contribute to identifying patients with potentially hazardous alcohol use among patients with different BP control in primary care. Ethical considerations remain essential in applying these methods.

## Supplementary Material

cmaf097_Supplementary_Data

## Data Availability

The data from this cohort are available upon reasonable request. Data access is governed by data sharing agreements that comply with relevant privacy and ethical guidelines.
