# Supplementary material for: Hypertension and alcohol: a cross-sectional study comparing PEth with AUDIT and AUDIT-C in primary care
Source: Fam Pract. 2026 Jan 9;43(1):cmaf097. doi: 10.1093/fampra/cmaf097 (PMC12787009; doi:10.1093/fampra/cmaf097)
Supplement: cmaf097_Supplementary_Data [file cmaf097_supplementary_data.pdf]

Supplementary file

Recruitment process

Table S1. Description of the recruitment process of 270 Swedish primary care patients with hypertension (ICD-10 code I10.9) in relation to blood pressure control (2022-2024), illustrated in flowchart (Figure 1) in manuscript.

	**Controlled hypertension**[1] n=4 102	**Uncontrolled hypertension**[2] n=2 756	**Apparent treatment resistant hypertension**[3] n=757
**Not checked for inclusion**	3 106	1 681	0
**Consecutively checked for inclusion**	996	1 075	757
**Excluded before invitation**			
Recent hypertension check-up, n	591	577	390
Other reasons:			
Not registered with Primary health care center, n	26	33	16
Other type of hypertension than ICD-10 I10.9, n	3	2	3
Not able to fill in questionnaire, n	20	19	4
Not able to visit PHCC, n	7	13	5
No diagnose of hypertension, n	1	1	
Age over 85 year, n	1		1
Unknown address and/or telephone number, n	1	3	1
**Total exclusions, n (%)**	650 (65)	648 (60)	420 (55)
**Total invited**	346	427	337
**Excluded after invitation**			
Declined, n (%)	224 (65)	297 (70)	240 (71)
Other reasons:			
Not registered with primary health care center, n	1	1	1
Other type of hypertension than ICD-10 I10.9, n			1
Recent annual hypertension check-up, n		2	
Not able to fill in questionnaire, n	3	4	8
Not able to visit PHCC, n	3	3	3
No diagnose of hypertension, n	3	2	
Study full, n	13	16	3
Unknown address and/or telephone number, n	2	5	5
**Total exclusions, n (%)**	249 (72)	330 (77)	261 (77)
**Included, n**	97	97	76
**Drop out, n**	1	2	2

[1]<140/90 mmHg, [2]≥140/90 mmHg, [3]≥140/90 mmHg with at least three antihypertensive medications regardless of class.

Patient Questionnaire[1]

We will be grateful if you fill in this patient questionnaire

Here are some questions about diseases. Please select one option per question.

1. **Do you have Type 1 diabetes?**
   - Yes
   - No
   - Don't know
2. **Do you have Type 2 diabetes?**
   - Yes
   - No
   - Don't know
3. **Do you have angina?** Also known as angina pectoris in Latin.
   - Yes
   - No
   - Don't know
4. **Do you have atrial fibrillation?**
   - Yes
   - No
   - Don't know
5. **Do you have heart failure?**
   - Yes
   - No
   - Don't know
6. **Have you had a heart attack?**
   - Yes
   - No
   - Don't know
7. **Have you had a stroke?** A stroke means a blood clot or bleeding in the brain.
   - Yes
   - No
   - Don't know
8. **Have you had a TIA?** A TIA means a blood clot in the brain that disappears after a short time.
   - Yes
   - No
   - Don't know

Here are some questions to help us understand your habits. Please select the answer that is most common for you. Select only one answer for each question.

1. **Do you smoke?**
   - No, I have never been a smoker
   - No, I quit smoking more than 6 months ago
   - No, I quit smoking less than 6 months ago
   - Yes, I smoke, but not every day
   - Yes, I smoke 1-9 cigarettes every day
   - Yes, I smoke 10-19 cigarettes every day
   - Yes, I smoke more than 19 cigarettes every day
2. **Do you use smokeless tobacco (Swedish snus)?**
   - No, I have never used Swedish snus
   - No, I quit using Swedish snus more than 6 months ago
   - No, I quit using Swedish snus less than 6 months ago
   - Yes, I use Swedish snus, but not every day
   - Yes, I use 1-3 cans every week
   - Yes, I use 4-6 cans every week
   - Yes, I use more than 6 cans every week
3. **Do you exercise on a level that makes you short winded, for example running, fitness class, or ball games?**
   - No, almost never
   - Yes, less than 30 minutes every week
   - Yes, 30-60 minutes every week
   - Yes, 60-90 minutes every week
   - Yes, 90-120 minutes every week
   - Yes, more than 120 minutes every week
4. **During a regular week, how much time are you physically active in ways that are not exercise, for example walks, bicycling, or gardening?** Add up all the times you move for at least 10 minutes.
   - No time
   - Less than 30 minutes every week
   - 30-60 minutes every week
   - 60-90 minutes every week
   - 90-150 minutes every week
   - 150-300 minutes every week
   - More than 300 minutes (5 hours) every week
5. **How often do you eat vegetables or root vegetables (fresh, frozen, or cooked)?**
   - Twice every day or more
   - Once every day
   - A few times a week
   - Once a week or less
6. **How often do you eat fruit or berries (fresh, frozen, canned, juice, etc.)?**
   - Twice every day or more
   - Once every day
   - A few times a week
   - Once a week or less

7. **How often do you eat fish or seafood (as a main course, in a salad, or as a sandwich-topping)?**
   - o Three times a week or more
   - o Twice a week
   - o Once a week
   - o A few times a month or less

8. **How often do you eat cakes, chocolate, sweets, crisps, or drink soft drinks?**
   - o Twice every day or more often
   - o Once every day
   - o A few times a week
   - o Once a week or less

9. **How often do you eat breakfast?**
   - o Every morning
   - o Almost every morning
   - o A few times a week
   - o Once a week or less

Here are some questions about your alcohol drinking habits. We appreciate if you answer them as accurately and honestly as possible by marking the option that applies to you. A "standard drink" means: 50 centilitres (cl) of medium-strong beer, 33 centilitres (cl) of strong beer, 12-15 centilitres (cl) of wine, 8 centilitres (cl) of strong wine, 4 centilitres (cl) of hard liquor.

1. **How often do you drink alcohol?**
   - o Never
   - o Once a month or less often
   - o 2-4 times a month
   - o 2-3 times a week
   - o 4 times a week or more

2. **How many "standard drinks" (see examples) do you drink on a typical day when you drink alcohol?**
   - o 1-2
   - o 3-4
   - o 5-6
   - o 7-9
   - o 10 or more

3. **How often do you drink six or more of these "standard drinks" on one occasion?**
   - o Never
   - o Less than once a month
   - o Monthly
   - o Weekly
   - o Daily or almost daily

4. **How often during the past year have you found that you were not able to stop drinking once you had started?**
   - o Never
   - o Less than once a month

- Monthly
- Weekly
- Daily or almost daily

5. **How often during the past year have you not done something you should have done because of drinking?**
    - Never
    - Less than once a month
    - Monthly
    - Weekly
    - Daily or almost daily
6. **How often during the past year have you needed a “drink” in the morning after a heavy drinking session the day before?**
    - Never
    - Less than once a month
    - Monthly
    - Weekly
    - Daily or almost daily
7. **How often during the past year have you had feelings of guilt or remorse after drinking?**
    - Never
    - Less than once a month
    - Monthly
    - Weekly
    - Daily or almost daily
8. **How often during the past year have you drunk so much that the next day you cannot remember what you said or did?**
    - Never
    - Less than once a month
    - Monthly
    - Weekly
    - Daily or almost daily
9. **Have you or someone else been injured because of your drinking?**
    - No
    - Yes, but not in the past year
    - Yes, during the past year
10. **Has a relative or friend, a doctor (or someone else in healthcare) been worried about your drinking or suggested that you should cut down?**
    - No
    - Yes, but not in the past year
    - Yes, during the past year

Here are some questions about your health. Please select only one option under each heading that best describes your health TODAY.

1. **Mobility**
    - I have no problems walking about
    - I have slight problems walking about

- I have moderate problems walking about
- I have severe problems walking about
- I am unable to walk about

2. **Self-care**
   - I have no problems washing or dressing myself
   - I have slight problems washing or dressing myself
   - I have moderate problems washing or dressing myself
   - I have severe problems washing or dressing myself
   - I am unable to wash or dress myself
3. **Usual activities** (e.g., work, study, housework, family-, or leisure activities)
   - I have no problems doing my usual activities
   - I have slight problems doing my usual activities
   - I have moderate problems doing my usual activities
   - I have severe problems doing my usual activities
   - I am unable to do my usual activities
4. **Pain/discomfort**
   - I have no pain or discomfort
   - I have slight pain or discomfort
   - I have moderate pain or discomfort
   - I have severe pain or discomfort
   - I have extreme pain or discomfort
5. **Anxiety/depression**
   - I am not anxious or depressed
   - I am slightly anxious or depressed
   - I am moderately anxious or depressed
   - I am severely anxious or depressed
   - I am extremely anxious or depressed
6. **We want to know how good or bad your health is TODAY.** This scale is numbered from 0 to 100. 100 is the best health you can imagine. 0 is the worst health you can imagine. Place a mark on the scale to show how your health is TODAY.

   Worst imaginable condition | 50 | Best imaginable condition

Here are some questions about your living conditions.

1. **What is your highest completed education?**
   - Compulsory school, elementary school, secondary school, or similar
   - Two-year upper secondary school or vocational school
   - 3-4 year upper secondary school
   - Folk high school or similar
   - University or university college, less than 3 years
   - University or university college, 3 years or longer
2. **What is your current employment status?**
   - Employed

- Self-employed
- On leave or parental leave
- Studying, internship
- Labour market measure
- Unemployed
- Retired
- Disability or activity compensation (early retirement or disability pension)
- Long-term sick leave (more than 3 months)
- Managing own household
- Other, please specify on the next line

3. **If your current employment status is employed: Indicate the percentage (%) of full-time.** Write in numbers, not words.

4. **If you indicated "Other" under Current employment status: Describe in your own words what your employment status is.**

5. **What is or was your main occupation or job?** If you are not working now, write the occupation or job you have mainly had. If you have multiple jobs, fill in the occupation or job that is your main one. Try to provide as detailed a description as possible. Instead of "teacher", write e.g. "primary school teacher". Instead of "nurse", write e.g. "district nurse". Instead of "driver", write e.g. "bus driver".

6. **Who do you live with?** This means those you live with at least half the time. If you mark "No one", you cannot mark any other boxes.
   - No one
   - Parents or siblings
   - Husband, wife, cohabitant, or partner
   - Other adults
   - Children

[1]English translation (from Swedish) by the authors.
